# An Unusual Combination of Acetabular and Pelvic Fracture: Is This a New Subtype of Acetabular Fracture?

**DOI:** 10.5812/traumamon.9613

**Published:** 2013-05-26

**Authors:** Reza Tavakoli Darestani, Gholamhosein Kazemian, Mohammad Emami Moghaddam, Alirea Manafi Rasi, Yadollah Alipour, Mohammad Mahdi Bagherian Lemraski

**Affiliations:** 1 Department of Orthopedics, Imam Hossein Medical Center, Shahid Beheshti University of Medical Sciences, Tehran, IR Iran

**Keywords:** Acetabulum, Fractures, Bone, Wounds and Injuries

## Abstract

**Introduction:**

Acetabular fractures are a common problem among young males. An acetabular fracture with disruption of the joint surface, if untreated, will rapidly lead to post-traumatic osteoarthritis. Proper reduction and internal fixation depend on accurate classification and the quality of imaging.

**Case Presentation:**

We present an unusual form of acetabular fracture, which is not included in the conventional classification (Judet and Letournel) ; this occurred in a middle-aged male who was operatively treated without any complications. In this case due to posterior extension of the fracture into the SI joint and concomitant anterior column fracture in the area above the acetabular dome, no portion of the acetabular anterior surface remained connected to the innominate bone.

**Conclusions:**

We recognized this type of fracture and treated it similarly to both column fractures. We recommend that the classification of acetabular fractures be modified to include this type of fracture.

## 1. Introduction

Acetabular fractures are life-altering injuries commonly occurring in young males who are active and productive members of the society. Orthopedic surgeons have used a variety of measures to determine patients’ outcome following acetabular fractures (1). The peak incidence of acetabular fractures is in the third decade of life (2). These fractures are usually the result of a high-energy trauma to the lower extremity, falling from heights or being hit by a car; they are often associated with head, visceral, spine and extremity trauma. An acetabular fracture with disruption of the joint surface, if untreated, will rapidly lead to post-traumatic osteoarthritis (3). Proper reduction and internal fixation depend on accurate classification and the quality of imaging (4). Three main goals of treatment include: Re-establishing a lasting and painless hip joint, early mobilization and avoidance of complications causing a poor outcome or the need for reoperation (5, 6). Two major groups of complications exist; the first group is mainly treatment dependent complications like infection, heterotopic ossification and implant failure (7, 8). The second group consists of post traumatic arthritis and sciatic nerve injury which may be related or unrelated to the treatment (9, 10). We present a case of unusual acetabular fracture with dome involvement extending into the SI joint and iliac wing coupled with anterior column fracture after a high energy trauma in a middle-aged male who was surgically treated without complications.

## 2. Case Presentation

A 34 year-old male was referred to our hospital with a pelvic trauma as a result of a fall from a 7m height. On physical examination the patient was unable to bear weight on the affected lower extremity. His pelvis was tender over the anterior superior iliac spine, the ischial tuberosity and the right sacroiliac joint. He also exhibited limited range of motion in the right hip. Neurovascular status was intact and the patient was hemodynamically stable. Clexane was administered at admission. The lumbar region, left hip and both knees had complete and painless range of motion.

### 2.1. Diagnosis

**Figure 1.**
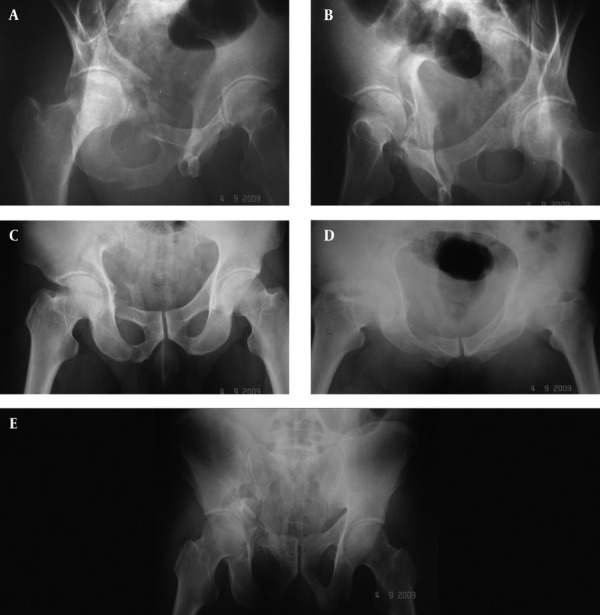
Preoperative Radiographs. A) Obturator oblique view indicates the fracture in the anterior acetabular column and superior and inferior rami of the pubis in the right hip; B) Iliac oblique view indicates the fracture line in the weight bearing dome extending to the SI joint without involving the posterior column; C) Anteroposterior view indicates the fracture line in superior articular surface extending to the SI joint; D) Inlet view indicates the fracture in the pubic rami; E) Outlet view indicates the posterior iliac wing and pubic rami fractures.

**Figure 2.**
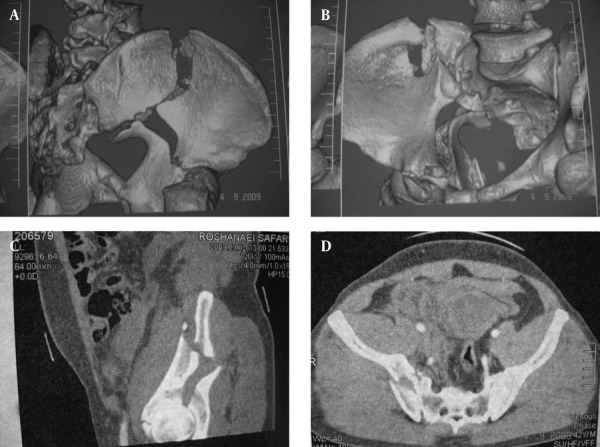
Preoperative CT Scans. A and B) Fracture of the iliac wing and fracture line in the weight bearing area of the SI joint is shown in the 3-D CTscan. Also note that the greater sciatic notch is separated without involvement of the posterior column; C and D) Sagittal and transverse views indicate the fracture of the iliac wing, superior articular surface and SI joint involvement.

The initial diagnosis of an acetabular fracture was made from the antero-posterior pelvic X-ray (Figure 1). Judet views and 3D CT demonstrated a fracture of the anterior column with a fracture line in the iliac wing and involvement of the SI joint (Figure 2). The fracture line in the weight bearing dome extended to the SI joint without involving the posterior column and this was an unusual type of acetabular fracture. In spite of continuity of the posterior column, the whole acetabulum was detached from the ilium, as the fracture entered the SI joint.

### 2.2. Surgery

**Figure 3.**
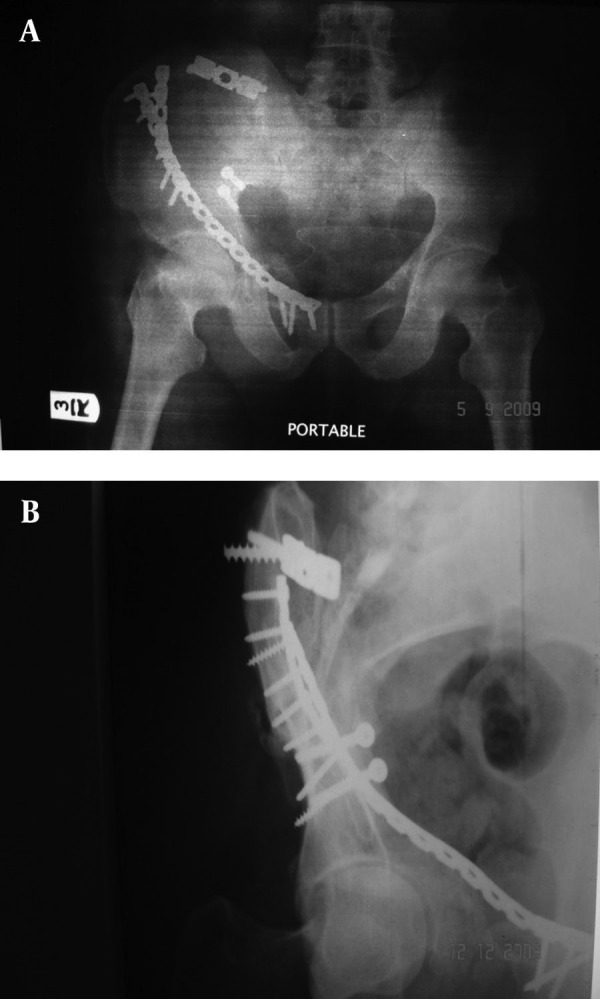
Postoperative Anteroposterior Pelvic View. A and B) The fracture was reduced and fixed using ilioinguinal approach with plates and screws

The patient underwent surgery 2 days after admission with open reduction and internal fixation of the acetabulum and iliac wing using an ilioinguinal approach. The acetabular fracture was fixed with a reconstructive plate and the posterior column was fixed using indirect reduction from the anterior column with 2 lag screws. Muscle strengthening and active range of motion exercises started 7 days postoperatively and full weight bearing was allowed 2 months after the operation. Post-operative X-rays (antero-posterior and Judet view of pelvis) were acceptable (Figure 3). There were no wound infections, iatrogenic nerve injuries or thromboembolic events. The patient walked without pain without functional limitations. The final radiographic results were excellent.

## 3. Conclusions

The fracture described in this case report cannot be classified using the Judet and Letournel (J-L) classification. Dome involvement in acetabular fractures is important and has a great impact on the type of treatment (non-surgical or surgical) and the outcome. In this case due to the posterior extension of the fracture into the SI joint and the accompanying anterior column fracture in the area above the acetabular dome no portion of the acetabular anterior surface remained connected to the innominate bone. This condition is similar to both column fractures in spite of continuity of the posterior column. Laflamme etal. have reported an isolated quadrilateral plate fracture of the pelvis with no involvement of either column but with an impaction fracture of the femoral head, which also cannot be classified according to the commonly used classification systems (11).In another case reported by Pascarella et al. an impacted fracture of the posteroinferior articular surface of the acetabulum with comminution of the quadrilateral lamina and fracture of the ischial bone has been discussed. In their case anterior and posterior columns were intact (12). This case also cannot be classified using the commonly used classification systems. We recognized this type of fracture and treated it similarly to both column fractures. We think it might be necessary to modify the Judet and Letournel classification to include unusual cases of acetabular fractures like the one discussed herein.
